# Inhibition of Dimethylarginine Dimethylaminohydrolase (DDAH) Enzymes as an Emerging Therapeutic Strategy to Target Angiogenesis and Vasculogenic Mimicry in Cancer

**DOI:** 10.3389/fonc.2019.01455

**Published:** 2020-01-09

**Authors:** Julie-Ann Hulin, Ekaterina A. Gubareva, Natalia Jarzebska, Roman N. Rodionov, Arduino A. Mangoni, Sara Tommasi

**Affiliations:** ^1^Clinical Pharmacology, College of Medicine and Public Health, Flinders University, Adelaide, SA, Australia; ^2^N.N. Petrov National Medical Research Center of Oncology, Saint Petersburg, Russia; ^3^Division of Angiology, Department of Internal Medicine III, University Center for Vascular Medicine, University Hospital Carl Gustav Carus, Technische Universität Dresden, Dresden, Germany; ^4^Department of Anesthesiology and Intensive Care Medicine, University Hospital Carl Gustav Carus, Technische Universität Dresden, Dresden, Germany

**Keywords:** DDAH, nitric oxide, ADMA, angiogenesis, vasculogenic mimicry, cancer

## Abstract

The small free radical gas nitric oxide (NO) plays a key role in various physiological and pathological processes through enhancement of endothelial cell survival and proliferation. In particular, NO has emerged as a molecule of interest in carcinogenesis and tumor progression due to its crucial role in various cancer-related events including cell invasion, metastasis, and angiogenesis. The dimethylarginine dimethylaminohydrolase (DDAH) family of enzymes metabolize the endogenous nitric oxide synthase (NOS) inhibitors, asymmetric dimethylarginine (ADMA) and monomethyl arginine (L-NMMA), and are thus key for maintaining homeostatic control of NO. Dysregulation of the DDAH/ADMA/NO pathway resulting in increased local NO availability often promotes tumor growth, angiogenesis, and vasculogenic mimicry. Recent literature has demonstrated increased DDAH expression in tumors of different origins and has also suggested a potential ADMA-independent role for DDAH enzymes in addition to their well-studied ADMA-mediated influence on NO. Inhibition of DDAH expression and/or activity in cell culture models and *in vivo* studies has indicated the potential therapeutic benefit of this pathway through inhibition of both angiogenesis and vasculogenic mimicry, and strategies for manipulating DDAH function in cancer are currently being actively pursued by several research groups. This review will thus provide a timely discussion on the expression, regulation, and function of DDAH enzymes in regard to angiogenesis and vasculogenic mimicry, and will offer insight into the therapeutic potential of DDAH inhibition in cancer based on preclinical studies.

## Introduction

Despite recent therapeutic advances, cancer remains among one of the leading causes of death worldwide, and the development of novel anti-tumor therapies is still a key priority. Clinical and experimental studies have documented the critical importance of an adequate blood supply for local solid tumor growth and distant metastasis (1–4). Furthermore, the ability of tumor cells to induce new blood vessel growth is a determining factor in both tumor size and spread. The process of angiogenesis, involving the formation, sprouting, extension, and remodeling of pre-existing blood vessels, is a well-accepted paradigm for the development of these intra-tumoral vascular networks (5, 6). Anti-angiogenic treatments for solid tumors have received much attention, yet studies have consistently revealed variable benefits among cancers of different origins. Positive results are often modest and not beneficial when long-term survival is considered (7–10).

Vasculogenic mimicry (VM) describes an alternative mechanism by which particularly aggressive tumors can acquire a micro-circulation: this process involves the formation of vessel-like networks lined by the tumor cells, effectively mimicking a true vascular endothelium (11–14). Not only does this process occur *de novo*, without the need for endothelial cells and independently of angiogenesis (15), but the tumor-lined vessels are also able to fuse to the conventional vascular network (16). There is evidence for VM networks in a number of cancers including those of the breast (17), prostate (18), brain (19), and ovaries (20, 21). The presence of these networks is generally predictive of poor survival and increased metastatic potential due to entrance of the tumor cells into the vasculature (17, 22–25). Intriguingly, the use of anti-angiogenic treatments may actually be a driving factor in the development of VM (26, 27), which may be at least partly induced by the resulting hypoxia (28). The presence of VM in cancers therefore represents a highly clinically relevant challenge both from a prognostic and a therapeutic point of view.

The signaling molecule nitric oxide (NO), a small short-lived free radical gas, has a fundamental role in diverse physiological processes across different tissues. Perhaps the most well-studied and established of these is its role in maintaining physiological homeostasis of the cardiovascular system. Research published simultaneously in 1987 by Ignarro et al. and Palmer et al. first identified NO as the endothelium-derived relaxing factor (29, 30). It is now clear that NO is not only a powerful vasodilator, central to the control of vascular tone, and blood pressure (31, 32), but is also critical for inhibition of platelet aggregation and promoting anti-inflammatory effects (33, 34). Importantly, NO is known to participate in vascular permeability and angiogenesis mediated by vascular endothelial growth factor (VEGF) (35). Due to the essential and diverse roles of NO, it is not surprising that altered NO concentrations result in significant pathophysiological conditions. These include numerous cardiovascular disorders, as well as neurodegenerative disorders, inflammatory arthritis, septic shock, schizophrenia, and various cancers, as previously reviewed (36).

The importance of NO in a range of cellular processes is further highlighted by its tight regulation at multiple levels, which is critical for both its spatial and dosage control. Endogenous NO is the product of a two-step redox reaction requiring molecular oxygen and a series of cofactors including flavin mono- and di-nucleotide, calmodulin, nicotinamide adenine dinucleotide phosphate, and tetrahydrobiopterin (37, 38). This biochemical synthesis of endogenous NO is governed by the family of nitric oxide synthase (NOS) enzymes through the stereospecific conversion of the natural amino acid L-arginine to L-citrulline and NO. The three distinct mammalian isoforms of NOS are NOS1 (also known as neuronal or nNOS), NOS2 (inducible or iNOS), and NOS3 (endothelial or eNOS), each exhibiting a unique expression pattern and named for their location of initial isolation; nNOS is predominantly expressed by resident cells of the central and peripheral nervous system including both neuronal and non-neuronal cells (39, 40), iNOS is expressed in inflammatory cells and can also be found in many other cell types in response to immunologic or inflammatory agents such as cytokines and lipopolysaccharides (41), and eNOS is predominantly expressed in endothelial cells. There is thus a regulation of NO synthesis that exists at the level of NOS transcription, post-translational modifications and specific cellular expression, as well as metabolic regulation at the level of NOS substrate availability (42). The activity of all three NOS isoforms is also regulated by the competitive inhibitors asymmetric dimethylarginine (ADMA) and monomethyl arginine (L-NMMA), which are ubiquitous endogenous metabolites of protein degradation that compete with the NOS substrate, L-arginine, for binding to the NOS active site (43–48). The two members of the dimethylarginine dimethylaminohydrolase (DDAH) family of enzymes, DDAH1 and DDAH2, are responsible for the degradation of the NOS inhibitors ADMA and L-NMMA (49) and are therefore key components in maintaining homeostatic control of NO.

There is a growing body of literature which demonstrates NO as a molecule of interest in carcinogenesis and tumor growth progression (50–52). In particular, dysregulation of the DDAH/ADMA/NO pathway, resulting in increased local NO availability, is often associated with promotion of tumor angiogenesis, growth, invasion, and metastasis. Increased expression of DDAH enzymes in tumors of different origins has been reported by numerous research groups in recent years, and inhibition of DDAH expression and/or activity in cell culture models and *in vivo* studies has indicated the potential therapeutic benefit of targeting this pathway (53–56). Additionally, whilst ADMA-mediated regulation of angiogenesis is highly relevant for tumor growth, DDAH enzymes may have dual ADMA-dependent and -independent effects on cancer progression. In this review we revisit the relevance of NO in cancer and provide an update in relation to cancer angiogenesis and VM. We also summarize a pioneering body of evidence for the potentially important expression, regulation, and function of DDAH enzymes in cancer initiation and/or progression. Finally, we discuss and offer insight into the therapeutic potential of DDAH inhibition as a cancer anti-angiogenic agent based on preclinical studies.

## Nitric Oxide as a Cellular Modulator of Angiogenesis

Nitric oxide (NO) is an endogenously and ubiquitously produced free radical gas that is readily able to permeate cell membranes due to its small size and high lipophilicity. The half-life of NO has been estimated to be within the range of 0.1–2s, thus allowing for rapid termination of NO signaling cascades following removal of the initial stimulus (57). Despite its short half-life, NO has a unique ability, as a result of its physicochemical properties, to diffuse over long distances (several 100 μ) within milliseconds. In addition, in contrast to conventional biosignaling molecules which act solely by binding to specific receptor molecules, NO manifests many of its biological actions via a wide range of chemical reactions. The precise reaction is dependent upon local NO concentration as well as composition of the extracellular and intracellular environment (58, 59). NO thus acts as a pleiotropic messenger, directly influencing a number of biological processes and pathophysiological conditions (36, 60).

The first physiological role identified for NO was its ability to bind and activate soluble guanylyl cyclase (sGC) in the cGMP signaling cascade (61); to date this remains the only known receptor for NO. Here, NO targets the heme component of sGC which allows for further coupling with cGMP-dependent protein kinase G, phosphodiesterases, and cyclic nucleotide gated channels (62, 63). In addition to inducing immune and inflammatory responses, this binding of NO to sGC mediates relaxation of smooth muscle and blood vessels, with a consequent increase in blood flow (64), prevents leukocyte adhesion and inhibits platelet aggregation thus maintaining vascular homeostasis and preventing atherosclerosis (65). Importantly, a number of studies indicate that NO is vital in promoting angiogenesis (66, 67). Angiogenesis is stimulated by NO production and attenuated when NO bioactivity is reduced, however the exact mechanisms underpinning these processes are complex.

NO is considered an “endothelial survival” factor as it inhibits apoptosis (68, 69) and enhances endothelial cell proliferation (70, 71), migration (67, 72), and podokinesis (73). These events are in part due to NO-mediated (primarily via eNOS and iNOS) increase in vascular endothelial growth factor (VEGF) or fibroblast growth factor expression (71, 74), and suppression of angiostatin production (75). There is a bidirectional interaction between VEGF and NO; VEGF can also promote NO synthesis via PI3 K/AKT-mediated phosphorylation of eNOS (76, 77). NO has also been identified as a regulator of isoforms of the antiangiogenic matricellular protein thrombospondin (TSP) through phosphorylation of extracellular signal-regulated kinase (ERK). Specifically, NO represses transcription of TSP2 (78), and triphasically regulates TSP1 protein expression dose-dependently (79). Furthermore, NO facilitates angiogenesis through stimulating the expression of matrix metalloproteinase (MMP). This is thought to be mediated by a cross talk between eNOS/iNOS and MMP via the VEGF receptor/cyclic adenosine monophosphate/protein kinase A/AKT/ERK signaling pathway. Consequently, ERKs upregulate the expression of membrane MMPs, thus favoring endothelial cell migration and vascular tube formation (80–82).

## The Dual Role of Nitric Oxide in Cancer

As synthesis of NO is generally a tightly regulated process, aberrant and dysregulated NO production is implicated in numerous pathophysiological conditions. It has been increasingly recognized that altered NO synthesis is associated with cancer initiation and progression, particularly cancer-driven angiogenesis, vasculogenic mimicry, and resulting metastasis. The dichotomous role of NO in cancer has been the subject of several reviews which highlight that NO can exhibit both oncogenic and tumor suppressing behavior depending on cancer type, location and stage, as well as local NO concentration and duration of exposure (50, 52, 83–87).

Modulation of NO concentration appears beneficial in mediating tumor regression and treatment for cancers characterized by reduced NO signaling, and this has been the focus of several research groups in recent years. An increase in NO concentration via the use of glyceryl trinitrate (GTN) reduced hypoxia-induced metastatic potential of an *in vitro* and *in vivo* model of murine melanoma (88) and exerted pro-apoptotic effects in colon cancer cell lines (89). Treatment with GTN has also shown potential for the treatment of prostate and small cell lung cancer by increasing sensitivity to chemotherapeutic agents (90–93). Similarly, the NO donor sodium nitroprusside has been demonstrated to suppress cell invasion in *in vitro* models of prostate and bladder cancer (94) and cell migration of gastric epithelial cells (95). Furthermore, it has shown protective effects due to apoptosis and growth inhibition in models of cervical cancer, pancreatic cancer, lymphoma, and glioma (96–99).

In contrast, other studies have demonstrated that excessive NO production is associated with poor prognosis and increased invasiveness of tumors of the breast (100–105) and with survival, proliferation and dedifferentiation of prostate cancer cells (106, 107). In head and neck cancer, excessive NO correlates with cancer risk and metastatic potential (52, 108, 109), and in colorectal cancer increased NO leads to enhanced angiogenesis and invasiveness (110, 111). Elevated NO concentrations have also been correlated with endometrial, cervical and gastric cancers, and tumors of the central nervous system (112–118). For these conditions, however, there is currently no targeted approach for intervention of NO production available for clinical use.

## DDAH Enzymes as Modulators of NO Synthesis

Together the NOS enzymes share 50–60% homology (119) and are all inhibited by asymmetrically methylated arginines (43–48). Methylarginines are endogenous metabolites of protein degradation and consist of monomethyl arginine (NMMA), asymmetric dimethylarginine (ADMA), and symmetric dimethylarginine (SDMA). They are continuously produced as the combination of two cellular processes: post-translational N-methylation of arginine residues incorporated into proteins, catalyzed by a family of protein methyltransferase (PRMT) enzymes (1–9) (120), and their subsequent release into the cytosol following proteolysis (121). Free methylarginines can then accumulate in the cytoplasm or cross cellular membranes where they are able to exert their biological function of inhibiting NOS enzymes in neighboring cells. Transport of methylarginines across cell membranes is typically controlled through transporters of the cationic amino acid (CAT) family, particularly CAT1, CAT2A, and CAT2B (122, 123). Both ADMA and NMMA inhibit all NOS isoforms, however plasma ADMA concentrations are considerably higher than those of NMMA (46, 124) and as such the relative contribution of NMMA to NOS inhibition has often been underestimated. Whilst ADMA and NMMA both compete with L-arginine for binding to the NOS active site (43–48), SDMA is not a direct inhibitor of NO synthesis. It can, however, reduce the availability of the NOS substrate L-arginine, by competing for transport by the CAT transporters (125).

**Figure 1:**
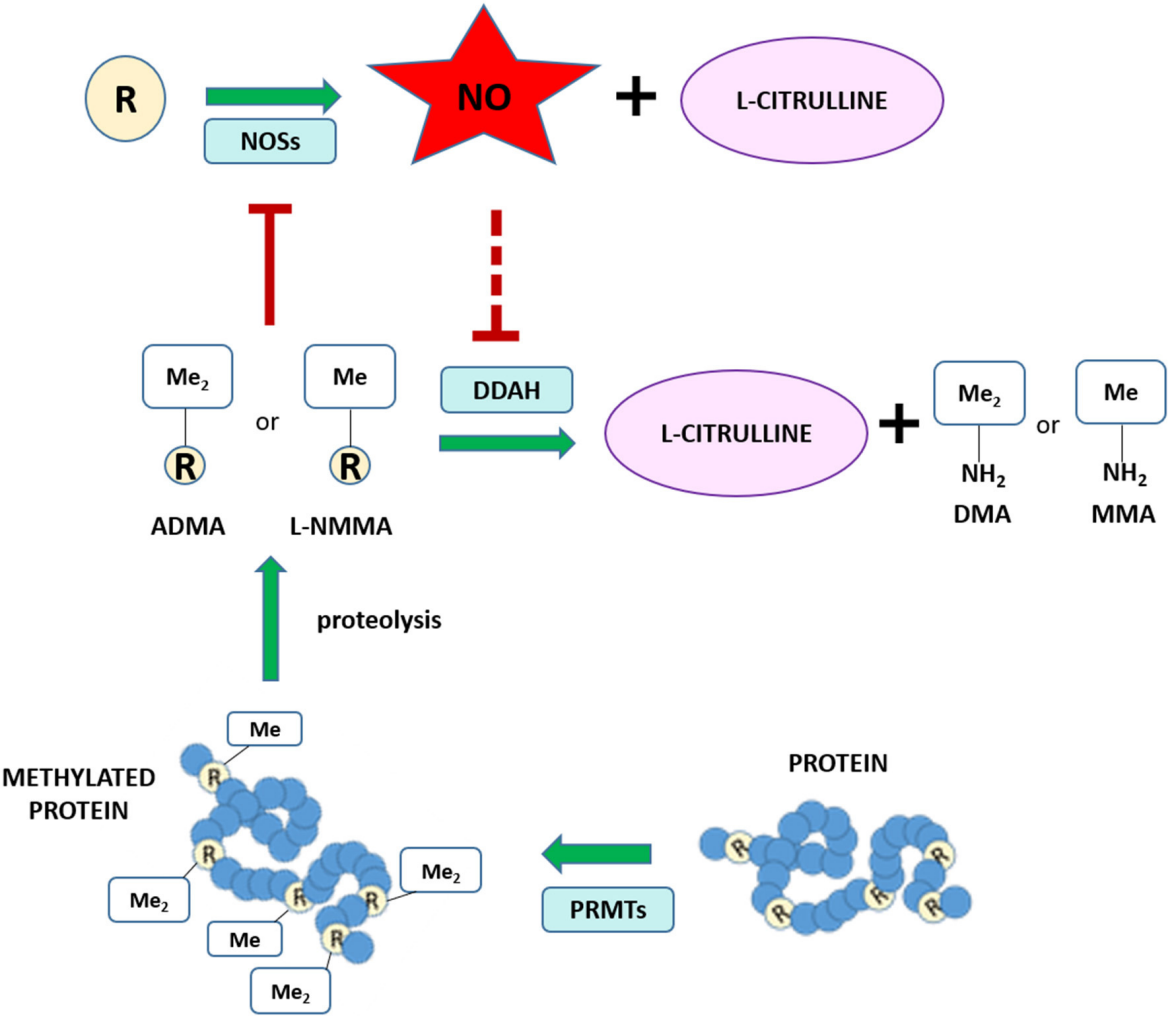
Schematic of the NO/ADMA/DDAH pathway. Nitric oxide (NO) is the product of the reaction catalyzed by the enzyme nitric oxide synthase (NOS). NOS catalyzes the conversion of the proteinogenic amino acid L-arginine (R) into NO and L-citrulline. All 3 isoforms of NOS (endothelial NOS, neuronal NOS and inducible NOS) are endogenously inhibited by asymmetrically methylated arginines (ADMA or R-Me_2_ and L-NMMA or R-Me). These endogenous inhibitors of the NO synthesis are generated and released in the cytosol as the product of 2 biomolecular processes: the post-translational methylation of arginine residues incorporated into proteins catalyzed by one members of the protein arginine methyltransferase (PRMT) family of enzymes and the release of said methylated residues into the cytosol by proteolysis. Methylated arginine can act as NOS inhibitors solely in their free form. The enzyme responsible for the metabolism of more than 70% of circulating and intracellular ADMA and L-NMMA is dimethylarginine dimethylaminohydrolase (DDAH), which converts ADMA and L-NMMA into L-citrulline and dimethylamine (DMA or Me_2_-NH_2_) or monomethylamine (MMA or Me-NH_2_).

Different routes of elimination have been identified for all three methylarginines. Two pathways for the metabolism of ADMA and SDMA are: (1) the transamination to asymmetric dimethylguanidinovaleric acid (ADGV) for ADMA and to symmetric dimethylguanidinovaleric acid (SDGV) for SDMA, mediated by alanine-glyoxylate aminotransferase 2 (AGXT2) (126–128), and (2) N-alpha-acetylation, although the enzyme responsible for catalyzing this reaction is still currently unknown (129–131). Conversion to γ-(dimethylguanidino) butyric acid has previously been proposed as a catabolic route for ADMA and SDMA (131), but the significance of this metabolic pathway has not received any further investigation. NMMA concentrations can also be regulated by the enzyme peptidylarginine deiminase 4 (PAD4), which catalyzes the deamination of NMMA residues still incorporated into proteins into L-citrulline (132). Renal excretion is responsible for the elimination of the majority of SDMA, but accounts for only a small percentage of ADMA clearance (<10% in some species) (131, 133–135). Most importantly, ADMA and NMMA are primarily metabolized by DDAH enzymes into L-citrulline and dimethylamine or monomethylamine, respectively (134, 136). The DDAH/ADMA/NO pathway is summarized in Figure 1.

Two DDAH isoforms have been identified in mammals (DDAH1 and DDAH2) and it is estimated that collectively more than 70% of ADMA is metabolized by these enzymes (137). Indeed, global heterozygous deletion of DDAH1 in mice increased plasma, brain, and lung ADMA concentrations by 20% (138). The DDAH isoforms are highly conserved at the amino acid level [62% in humans (49, 139)], particularly with residues important for substrate binding and hydrolysis. DDAH isoforms are also highly conserved across species, with high homology between the human, mouse, rat, and bovine gene sequences (DDAH1: 92%, DDAH2: 95%). While researchers are in agreement with DDAH1 being the key enzyme responsible for ADMA and NMMA metabolism (94, 140), there is conflicting evidence surrounding the metabolic activity of DDAH2.

Several lines of evidence suggest that under normal conditions DDAH1 is the isoform responsible for ADMA metabolism (141). Firstly, the tissues from DDAH1 KO mice do not display any DDAH activity (140). Secondly, silencing of DDAH1 in cultured vascular endothelial cells results in ADMA accumulation and a decrease in NO production, while silencing of DDAH2 has no effect (140). Consistent with this finding, overexpression of DDAH1 in cultured endothelial vascular cells decreases ADMA content and overexpression of DDAH2 does not (142). Purified recombinant DDAH2 was originally reported to metabolize NMMA (49) but following studies have failed to reproduce the metabolic activity of DDAH2 *in vitro* (143). Fluctuations in ADMA concentrations are observed in response to over-expression and/or knockout of the DDAH2 gene (144–146), but whether DDAH2 affects ADMA concentration *via* direct metabolism or by indirect regulation of its metabolism still remains unclear. The difficulties in recapitulating DDAH2 activity *in vitro* may suggest the requirement for additional cofactors or protein-protein interactions, or a missing step in the pathway of ADMA metabolism that is not functional in the cell lysates often used to assess recombinant DDAH2 protein function. Regardless, based on current available knowledge the DDAH1 enzyme appears to be key for metabolism of ADMA/NMMA and thus more relevant in regard to the treatment of cancer through the ADMA/NO pathway.

### Implications for Angiogenesis

The DDAH enzymes play a key role in homeostasis of the cardiovascular system, and specifically in modulation of angiogenesis and neovascularization. Whilst it appears that the majority of DDAH function is attributed to degradation of ADMA and thus modulation of NO synthesis, ADMA-independent functions of DDAH have also been identified.

ADMA plays a key inhibitory role in the formation of new blood vessels; examples include inhibition of proliferation of bovine retinal capillary endothelial cells (147) and coronary artery endothelial cells (148). Furthermore, *in vitro* and *in vivo* studies show that ADMA modulates all the key aspects of VEGF-induced angiogenesis: activation, proliferation, differentiation, and migration of endothelial cells. Fiedler and colleagues demonstrated that increased ADMA concentrations inhibit the VEGF-induced capacity of human umbilical vein endothelial cells (HUVECs) to form tubes on Matrigel by disrupting chemotaxis, migration, protrusion formation, focal adhesion turnover and reducing cell polarity and gap junction intercellular communication (74). In the same study, ADMA was also reported to interfere with activation of Rho GTPases via RhoA activation and Rac1 and Cdc42 inhibition. By inhibiting NO synthesis, ADMA reduced VEGF-mediated phosphorylation of VASP and Rac1 activation in human endothelial cells (74). This is consistent with what has been previously observed in pulmonary endothelial cells (149). Moreover, it appears that ADMA can interfere with the activation of endothelial progenitor cells (EPCs) (150). ADMA supplementation has also been reported to accelerate high glucose-induced EPC senescence, whilst the opposite effect was observed with the overexpression of DDAH2 (151). This is in line with association studies showing an inverse correlation between the number of EPCs in blood and plasma ADMA levels in coronary artery disease (150), peripheral arterial disease (152), and after renal transplantation (153). Additionally, increased plasma ADMA concentrations are linked to higher cardiovascular risk and numerous vascular diseases, many of which are associated with low NO output and endothelial dysfunction (154–158).

The generation of heterozygous DDAH1 knockout mice by Leiper and colleagues first demonstrated that DDAH1^+/−^ mice exhibited accumulation of ADMA and reduced NO concentrations, leading to vascular pathophysiology such as endothelial dysfunction, structural alterations in the pulmonary vasculature and decreased heart rate and cardiac output (138). Importantly, angiogenesis was significantly reduced in these mice, as assessed by quantification of microvessels sprouting from aortic rings (149) and hemoglobin content in plugs (74). Over-expression of DDAH1 reversed the anti-angiogenic effects associated with increased ADMA (74). The more recent generation of global DDAH1 deficient mice further confirmed the importance of DDAH1, but not DDAH2, for ADMA metabolism and in cardiovascular physiology (140). DDAH1^−/−^ mice exhibit impaired endothelial cell proliferation and decreased neovascularization (142). The generation of an endothelium-specific DDAH1^−/−^ mouse using Tie-2 driven Cre expression demonstrated that intracellular ADMA concentrations are crucial in determining the endothelial cell response. Whilst the angiogenic response was significantly impaired both *in vivo* and *ex vivo*, plasma ADMA concentrations, vasoreactivity *ex vivo* and hemodynamics *in vivo* remained unaffected (159). Together, these studies further highlight the essential role of DDAH1 in ADMA and NMMA metabolism.

The expression and activity of both DDAH1 and DDAH2 appear to be critical for wound healing and angiogenesis. Overexpression of DDAH1 in endothelial cells resulted in enhanced tube formation when grown on Matrigel and an increase in VEGF mRNA expression; blocking DDAH activity reversed these effects (160). DDAH1 overexpressing mice exhibited enhanced neovascularization after hind limb ischemia (161, 162) and improved endothelial cell regeneration with reduced neointima formation following vascular injury (163). Conversely, DDAH1 knockout mice had reduced endothelial repair and angiogenesis, and impaired endothelial cell proliferation compared with WT mice in a model of carotid artery wire injury. Interestingly, VEGF-expression was reduced in this DDAH1 global KO mouse model via a mechanism that was independent from the NO/cGMP/PKG pathway, and regulated by the Ras/PI3K/Akt pathway (142). In fact, experiments performed in DDAH^−/−^ mice (142), siRNA-mediated DDAH1 knockdown and DDAH1 overexpressing HUVEC cells (164) have demonstrated that DDAH1 regulates HUVEC cell cycle progression via Ras/Akt activation and modulation of cyclinD1, cyclinE, CDC2, and CDC25C concentrations. Moreover, DDAH1 was reported to regulate angiogenesis by increasing NO concentrations, which induces caspase-3 activation in human fetal pulmonary microvascular endothelial cells (165). Increased angiogenesis is also observed following transfection of endothelial cell lines with DDAH2 (166), a process that is partially mediated by the upregulation of VEGF expression through a Sp1-dependent and NO-independent mechanism (167). Furthermore, comparative studies performed in DDAH1^+/−^, DDAH2^+/−^, and DDAH2^−/−^ mice have demonstrated the important role of DDAH2 in pathogenic retinal ischemia and ischemia-induced angiogenesis and the protective potential of DDAH2 inhibition against aberrant neovascularization (146). It seems that this is achieved through reduced ADMA metabolism and improved vascular regeneration in a VEGF-independent fashion. Another ADMA-independent mechanism by which DDAH2 appears to regulate angiogenesis involves the regulation of VEGF and kinase-domain insert containing receptor (KDR) expression within the silent information regulator 1 (SIRT1) pathways in EPCs (168).

Taken together, these studies demonstrate the key role of the DDAH/ADMA pathway in the regulation of neovascularization and endothelial cell proliferation, differentiation, and motility *in vivo* and *in vitro*. Impairment of the DDAH/ADMA/NO pathway and subsequent endothelial dysfunction have been extensively studied in relation to cardiovascular and renal disorders. The importance of the DDAH enzymes in cancer angiogenesis, neovascularization, and vasculogenic mimicry has only recently begun to be unraveled.

## Expression and Regulation of the DDAH Enzymes

### DDAH Expression

Whilst synthesis of ADMA occurs in all cells, expression of DDAH isoforms is variable. The two DDAH isoforms (DDAH1 and DDAH2) display distinct but overlapping tissue distribution, and additionally show some overlap with the constitutively expressed NOS isoforms. DDAH2 is expressed in the heart, vascular endothelium, kidney, placenta, and adipose tissue (169, 170). Sites of DDAH1 expression are considerably wider, but it is predominantly found within the brain, liver, and kidney (140, 171–176), the organs which represent the major sites of ADMA metabolism (141, 177, 178), as well as in the heart, lung, skeletal muscle, nervous system, spinal dorsal horn, and trophoblasts (138, 179–181). It is also important to mention that the expression pattern of DDAH1 and DDAH2 does not necessarily reflect the tissue activity of the enzymes. This issue is further complicated by the fact that the currently available DDAH activity assays do not distinguish between DDAH1 and DDAH2 isoforms. Therefore, even if two tissues display the same level of DDAH activity, it is unclear what amount of activity can be attributed to each DDAH isoform. This could be of particular importance given the additional ADMA-independent effects of both enzymes, as discussed later.

### Expression of DDAH Is Altered in Cancer

**Figure 2:**
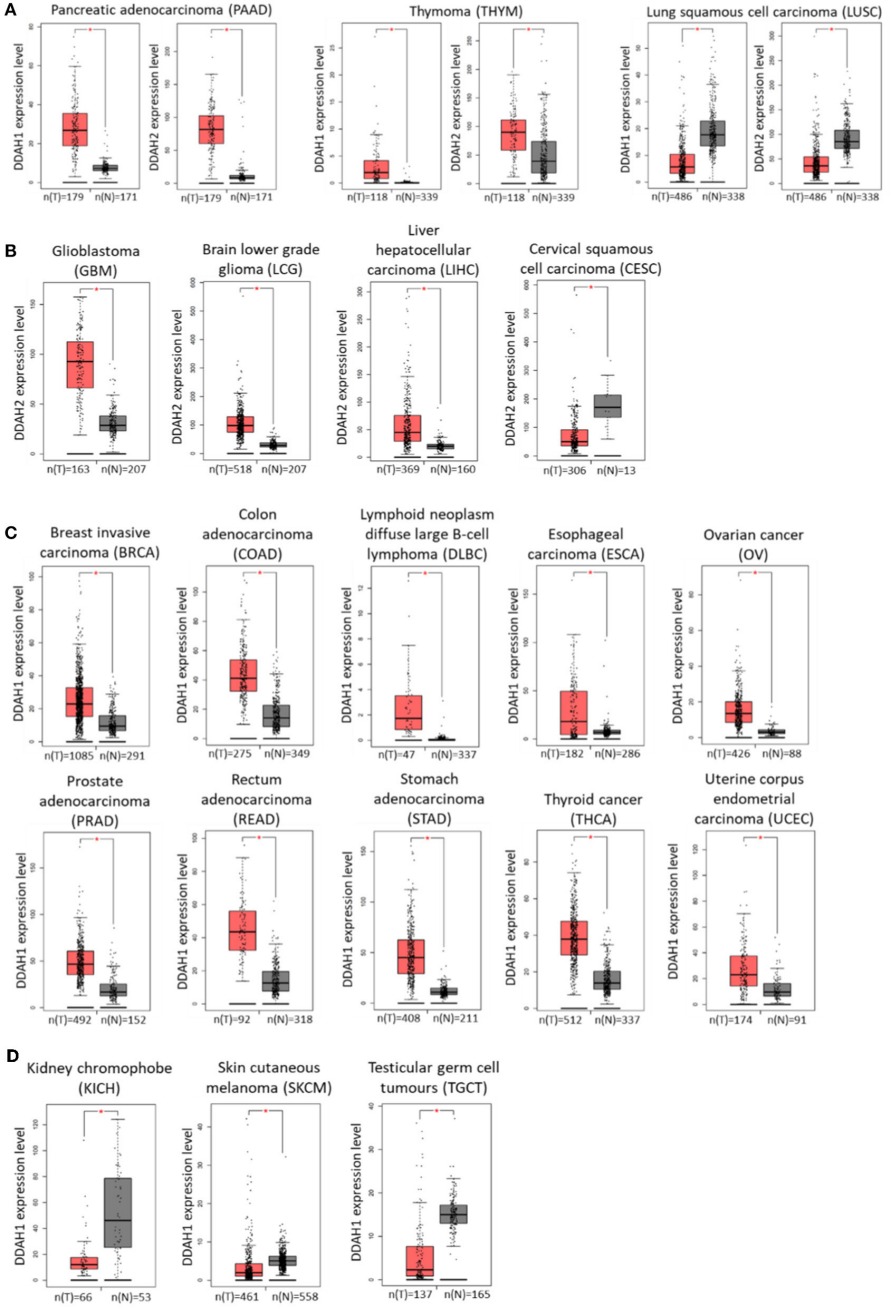
DDAH1 and DDAH2 transcript expression in various cancer tissues, determined from publicly available data generated by The Cancer Genome Atlas (TCGA) Research Network (http://cancergenome.nih.gov) and the Genotype-Tissue Expression (GTEx) project. Gray bars (N) denote normal tissue and red bars (T) denote corresponding tumor tissue. **(A)** DDAH1 and DDAH2 expression are both significantly altered in the same direction. **(B)** DDAH2 expression is significantly altered in tumor tissue. **(C)** DDAH1 expression is significantly increased in tumor tissue. **(D)** DDAH1 expression is significantly decreased in tumor tissue. Graphs were generated by the online web-tool Gene Expression Profiling Interactive Analysis (GEPIA; http://gepia.cancer-pku.cn/) (182).

Identification of genes which are differentially expressed in cancer relative to normal tissue can be highly beneficial in terms of developing new diagnostic, prognostic, and targeted therapeutic treatments for cancer development and progression. The recent advances in genome-wide transcriptomic and proteomic techniques have allowed for profiling of different cancers at various disease stages with this aim in mind. Interrogation of publicly available data generated by The Cancer Genome Atlas (TCGA) Research Network (http://cancergenome.nih.gov) and the Genotype-Tissue Expression (GTEx) project identified altered expression of DDAH1 and DDAH2 in various cancer tissues. The online web-tool Gene Expression Profiling Interactive Analysis (GEPIA; http://gepia.cancer-pku.cn/) (182) was used to analyse RNA-seq expression data sourced from these databases and to generate expression profiles of DDAH mRNA expression in comparable normal and tumor tissues for each cancer type. In pancreatic adenocarcinoma and thymoma DDAH1 and DDAH2 mRNA is significantly increased, whilst both DDAH1 and DDAH2 expression is decreased in lung squamous cell carcinoma (Figure 2A). Interestingly, with the exception of these three cancers, the expression of DDAH1 and DDAH2 does not change in the same direction. Instead, the expression of either isoform is altered independently of the other. There is also no evidence for an inverse correlation of DDAH1 and DDAH2 expression (e.g., an increase in DDAH1 expression paired with a decrease in DDAH2 expression, or vice versa) in any cancer type for which data is available in the TCGA database.

An increase in DDAH2 mRNA expression is further observed in glioblastoma, brain lower grade glioma and liver cancer samples, whilst a decrease is observed in cervical cancer samples relative to normal tissue (Figure 2B). The range of cancer types that display altered DDAH1 expression is significantly broader than that for DDAH2. In the majority of cases where there is a change in DDAH1 expression in tumor samples, it is significantly increased: these include breast cancer, colorectal cancer, lymphoid neoplasm diffuse large B-cell lymphoma, esophageal cancer, ovarian cancer, prostate cancer, rectal adenocarcinoma, stomach cancer, thyroid cancer, and uterine corpus endometrial carcinoma (Figure 2C). A decrease in DDAH1 expression is only found in chromophobe renal cell carcinoma (a rare form of kidney cancer), melanoma and in testicular germ cell tumors (Figure 2D). The relative expression of DDAH1 and DDAH2 mRNA in various cancers, and the sample number for each analysis, is shown in Figure 2.

In addition to RNA-seq data obtained through mining of TCGA datasets, a number of research groups have also identified altered DDAH mRNA and protein expression in various cancer cell lines and cancer tissues (Table 1). Studies to date have demonstrated an increase in DDAH1 protein expression in human glioma, meningioma, prostate cancer, and hepatocellular carcinoma, primarily by means of large-scale proteomic analysis. An upregulation of DDAH1 protein has also been observed in cohorts of melanoma and breast cancer cell lines, relative to normal melanocyte, and mammary epithelial cells, respectively (183, 187). Aside from the identification that DDAH1 expression is significantly altered in these cancers, only a handful of these studies undertook further analysis into the specific role and function of DDAH1 within each cancer context.

**Table 1:** DDAH1 and DDAH2 expression in human cancer tissues and cell lines.

**Sample**	**Method**	**DDAH1**	**DDAH2**	**References**
Melanoma cell lines	WB, IHC	↑^*^	↔	(183)
Prostatic cancer cell line (vs. benign prostatic hyperplasia cells)	WB	↔	↑	(184)
Prostate cancer tissue	Proteomic	↑	↔	(185)
Prostate cancer tissue (vs. normal and benign tissue)	TMA	↑	ND	(54)
Prostate cancer metastasis-derived prostasomes	Proteomic	↑	↔	(186)
Breast cancer cell lines (vs. normal mammary epithelial cells)	WB, qRT-PCR	↑	ND	(187)
Hepatocellular carcinoma (vs. non-tumorous liver)	WB, IF	↑	ND	(188)
Cerebrospinal fluid, serum, urine from patients with diffuse intrinsic pontine glioma	Proteomic, IHC, WB	↑	↔	(189)
Meningioma tissue (aggressive vs. indolent)	Proteomic, WB	↑	↔	(190)
Merkel cell carcinoma tissue (poor vs. good prognosis)	Transcriptomic	↑	↔	(191)
Pancreatic carcinoma tissue	Transcriptomic	↓	↔	(192)
Gastric cancer tissue and cell lines	IHC, WB, qRT-PCR	↓	ND	(193)
Lung adenocarcinoma	IHC, ISH, WB	ND	↑ (cancer associated fibroblasts)	(166)

*↑, increased expression; ↓, decreased expression; ↔, no change in expression; ND, not determined; WB, western blot; IHC, immunohistochemistry; TMA, tissue microarray analysis; qRT-PCR, quantitative realtime-PCR; IF, immunofluorescence; ISH, in situ hybridization*.

* *Upregulated in 78% of cell lines investigated*.

In addition to protein expression, Buijs et al. (188) further assessed DDAH1 catalytic activity in hepatocellular carcinoma (HCC) tissue relative to paired non-tumorous liver tissue. In tissue homogenates, mass spectrometry analysis of arginine and ADMA concentrations revealed a 74% increase in the arginine:ADMA ratio, which is indicative of increased ADMA metabolism and thus increased NO production. Furthermore, increased NO concentration was predicted in both tissue homogenates and serum from preoperative HCC patients, as measured by NO metabolites (nitrate and nitrite) using a colorimetric Griess assay. An increase in expression of the angiogenesis stimulating factor, VEGF, was also observed in HCC tissue samples. It is important to note that immunofluorescence analysis of tumor tissue samples confirmed expression of DDAH1 localized to hepatocytes, and absent from neighboring endothelial cells of vascular structures (188). We have also recently published evidence for a novel role of DDAH1 in breast cancer, particularly in the more aggressive and invasive triple negative breast cancer (TNBC) molecular subtype (187). In this study we demonstrated high expression of functional DDAH1 enzyme in TNBC cells relative to normal mammary epithelial cells. This was determined by both western blot analysis and mass spectrometry assessment of L-citrulline formation with 200 μM ADMA substrate. Inhibition of DDAH1 protein expression in these cells resulted in reduced L-citrulline formation, increased intracellular ADMA concentration and a reduced arginine:ADMA ratio; all consistent with decreased ADMA metabolism and consequently decreased NO production (187).

In 2011, a proteomics and pathway analysis study by Ummanni et al. identified DDAH1 overexpression in histologically characterized prostate cancer tissue, and highlighted its potential as a novel biomarker for prostate cancer development and/or progression (185). Intriguingly, whilst western blotting validated dysregulation of DDAH1 protein in tumor tissue, no significant change in DDAH was observed at the mRNA level. This is somewhat consistent with data in breast cancer cell lines, where a much greater change in DDAH1 expression was observed at the protein level compared to the transcript level (187). It is possible that this phenomenon is in part due to post-transcriptional regulation of DDAH1, likely mediated by multiple microRNA regulators in the unusually long DDAH1 3′UTR (2,971 bp). In a recent follow-up study, tissue microarray analysis further confirmed higher DDAH1 expression in prostate cancer compared to benign prostatic hyperplasia and normal prostate tissues; the expression of which correlates well with the aggressiveness of prostate cancer and suggests its role in disease progression (54). In hormone-dependent (PC3) and hormone-independent (LNCaP) prostate cancer cell lines, both of which express DDAH1, generation of L-citrulline from the enzyme-substrate ADMA was observed in a colourimetric assay. In alignment with findings in breast cancer cell lines (187), specific knockdown of DDAH1 protein in PC3 and LNCaP cell lines not only resulted in reduced L-citrulline formation, but also significantly increased intracellular ADMA concentration and decreased NO metabolite concentration.

In contrast to these studies, DDAH1 protein downregulation was frequently detected in gastric cancer tissues, where its low expression was associated with more lymph node metastasis and poorer clinical outcome (193). Knockdown and overexpression of DDAH1 in gastric cancer cell lines recapitulated these findings: cells overexpressing DDAH1 migrated more slowly and were less invasive *in vitro*, and displayed decreased metastatic potential *in vivo*, possibly through inhibition of epithelial-mesenchymal transition (EMT) pathways (193). The authors also reported reduced β-catenin expression following DDAH1 overexpression, and suggested that DDAH1 mediates β-catenin degradation via the Wnt signaling pathway, thus inhibiting EMT. The exact mechanism by which DDAH1 modulates β-catenin expression is currently undefined; there was no assessment of DDAH1 catalytic activity and subsequent NO production in this study. To the best of our knowledge, this represents the only study to date that identifies DDAH1 as a tumor suppressor. It is possible that the tumor suppressor role of DDAH1 in gastric cancer is independent of its role in the ADMA/NO pathway.

DDAH2 protein expression has been less extensively studied in cancer, but an upregulation has been reported in prostate cancer cell lines as well as the malignant stroma (but not tumor cells) of non-small-cell lung cancer tissue (166, 184). In the LNCaP prostate cancer cell line DDAH2 was more strongly expressed when compared to benign prostate hypertrophy cells, and was also accompanied by increased eNOS, iNOS, and VEGF expression (184). It is likely that a combination of these factors, and not specifically DDAH2 expression, is responsible for the increased NO production that was observed in these cells. Interestingly, the NOS inhibitor *N*^G^-nitro-L-arginine methyl ester (L-NAME), which is not degraded by DDAH, significantly increased DDAH2 expression and elevated NO production (184). A more recent study in 2016 identified increased expression of DDAH2 in the stroma fibroblasts of lung adenocarcinomas, where tumors with high stromal DDAH2 expression had a poorer prognosis (166). Almost all cases of minimally invasive adenocarcinoma and invasive adenocarcinoma were positive for DDAH2, while only half of pre-invasive lesions (atypical adenomatous hyperplasia and adenocarcinoma *in situ*) were positive. In contrast, in normal lung tissue only the vascular endothelium showed staining for DDAH2 (166).

### DDAH Regulation

Regulation of both DDAH1 and DDAH2 expression and activity is mediated via various mechanisms at different levels.

#### Post-translational Modulators of DDAH Activity

DDAH exists as a holoenzyme bound to a single inhibitory zinc ion. Removal of the zinc by either phosphate or imidazole results in increased DDAH enzymatic activity, thus demonstrating the regulatory role that the zinc binding site plays (194). The crystal structure of DDAH1, purified from bovine brain, shows zinc bound to the active site cysteine (Cys273); 95% of total DDAH1 purified protein exists as the zinc-bound form. These data suggest DDAH1 exists predominantly in its inhibited conformation (195). NO itself is a reversible inhibitor of DDAH activity through S-nitrosylation of the active site cysteine residue (Cys273 in bovine DDAH1, Cys274 in human DDAH1, Cys249 in human DDAH2), which involves covalent attachment of nitrogen monoxide to the thiol chain of the specific cysteine residues. Typically, this is associated with increased expression of iNOS and thus increased NO synthesis, and does not occur under basal conditions (196). It has been demonstrated *in vitro* via incubation of purified bovine DDAH or recombinant bacterial DDAH with a NO donor (DEA NONOate; 2-(*N,N-*dimethylamino)-diazenolate-2-oxide) (197, 198). This represents a feedback loop whereby subsequent accumulation of the DDAH substrates, ADMA and L-NMMA, in turn reversibly inhibit the NOS enzymes. Intriguingly, NO-induced DDAH inhibition is significantly more potent in the absence of zinc (DDAH apo-enzyme), which suggests zinc binding is protective of DDAH S-nitrosylation (198). Phosphorylation of rat DDAH1 at Ser33 and Ser56 has been reported (199), however the impact of this on DDAH1 activity is currently unknown. There is currently no further evidence to suggest additional posttranslational modification of DDAH enzymes.

There are a significant number of endogenous compounds, vitamins, and therapeutics identified to date that act as DDAH activators or inhibitors without altering gene expression. Many of these factors modulate DDAH activity via oxidative effects, such as via attenuation of low-density lipoprotein-induced endothelial dysfunction or by induction of reactive oxygen species. Key examples include 17β-estradiol (200), insulin (201), vitamin E (202), and the antioxidant Probucol (203) as DDAH activators. In contrast, the cytokine TNF-α (204), glucose (201, 205), s-nitrosohomocysteine (206), and erythropoietin (207) are significant inhibitors of DDAH activity. With the exception of s-nitrosohomocysteine, the exact mechanisms by which these compounds function to modulate DDAH activity is as yet undefined, however literature suggests that ultimately it is S-nitrosylation of DDAH and/or a modulation of zinc availability or binding capacity to the DDAH active site which are likely contributors. For example, induction of DDAH enzymatic activity may require a zinc-binding protein to act as a zinc receptor, thus abolishing the zinc-mediated inhibition of DDAH. On the other hand, zinc released from a redox sensitive zinc-binding protein, under conditions of oxidative or nitrosative stress, may bind to and inactivate DDAH. A recent study by Bollenbach and colleagues has also identified a DDAH inhibitory role for some naturally occurring amino acid derivatives, namely NG-hydroxy-L-arginine, Nω,Nω-dimethyl-L-citrulline and connatin (208).

Taken together, the number and diversity of endogenous compounds, vitamins, and therapeutics which are capable of altering DDAH activity highlights the importance of quantifying DDAH activity in tissues of interest. As protein expression may not necessarily reflect enzyme activity, a comprehensive understanding of the importance of DDAH enzymes in any given tissue or disease state requires assessment of transcript abundance, protein expression, and additionally activity of DDAH enzymes.

#### Transcriptional and Post-transcriptional Regulation of DDAH Expression

The understanding of what regulates DDAH expression in cancer is very limited. The only study to specifically address regulation of DDAH1 in cancer was performed in breast cancer cell lines and identified the microRNA miR-193b as a direct negative regulator through the DDAH1 3′UTR (187). In MDA-MB-231 cells expressing endogenous DDAH1, ectopic expression of miR-193b reduced DDAH1 mRNA and protein expression and decreased the conversion of ADMA to citrulline. Conversely, inhibition of miR-193b in the MCF7 cell line, which was absent for DDAH1 expression, was sufficient to induce DDAH1 (187). Mir-193b has been previously reported as a tumor suppressor in breast cancer tissues (209, 210) and is frequently downregulated in other solid tumors such as melanoma (211), liver cancer (212), and prostate cancer (213), all of which are reported to exhibit increased DDAH1 expression (Table 1). It is therefore plausible that miR-193b is an important regulator of DDAH1 expression in multiple cancers.

The DDAH1 3′UTR is unusually long (2,971 bp) and is therefore likely regulated by multiple microRNAs. In addition to miR-193b, various studies have demonstrated direct regulation of DDAH1 by miR-21 (214–216); however all studies to date have been performed in human endothelial cells. miR-21 was one of the earliest defined oncomiRs, and its role in carcinogenesis has been thoroughly investigated (217), particularly in gastric cancer where it is often upregulated (218, 219). In alignment with this, downregulation of DDAH1 is reported in gastric cancer tissue and cell lines (193). In HUVECs, transmembrane glycoprotein neuropilin-1 increases DDAH1 expression, mediated by a post-transcriptional mechanism involving miR-219-5p (220). Although this regulation has not been assessed in cancer, miR-219-5p has been reported to have a tumor suppressive role in colon cancer (221, 222) and ovarian cancer (223), which may in part relate to regulation of DDAH1.

Further studies on regulation of DDAH1 have identified that DDAH1 protein is increased in a time- and dose-dependent manner in cultured rat smooth muscle cells stimulated with IL-1β (224), and that O subfamily of forkhead (FoxO)1 is pivotal in regulation of endothelial activation as a negative regulator of DDAH1 (225). Agonists of the nuclear receptor farnesoid X receptor (FXR) have been shown to induce hepatic DDAH1 transcription through a promoter FXR response element, resulting in decreased plasma ADMA (172). Another study has also reported an increase in DDAH1 following stimulation with an FXR agonist in the liver and kidney, which was also accompanied by decreased plasma ADMA (226). Activation of FXR with bile acids has been found to enhance tumor angiogenesis (227), however whether FXR alters DDAH1 expression in cancer cells has yet to be identified. Furthermore, metal-responsive factor 1 (MTF1), a pluripotent transcriptional regulator induced by various stress conditions such as hypoxia and oxidative stress, increases DDAH1 expression *via* a direct binding site in the DDAH1 promoter (228). Hypoxia, which is often observed in solid tumors, induced DDAH1 expression in liver cancer HepG2 cells (188), however the exact mechanism underlying this induction remains to be elucidated.

The promoters of both DDAH1 and DDAH2 contain sterol response elements (DDAH1 more so than DDAH2). In cultured endothelial cells, the sterol response element binding protein (SREBP) transcription factor member, SREBP-2, was found to bind the DDAH1 promoter and activate transcription (229); knockdown of SREBP-2 led to a decrease in DDAH1 mRNA expression. SREBPs are key transcription factors which play a central role in lipid metabolism, and elevated SREBP levels are common in various cancers (230, 231). It appears that regulation by SREBPs is isoform-specific, however, as SREBP-1c decreased both DDAH1 and DDAH2 expression (229). Finally, an increase in DDAH activity in human and murine endothelial cell lines has been demonstrated following treatment with estradiol (200). In following studies, an estrogen receptor (ER) binding site was identified within the DDAH2, but not the DDAH1, promoter (232), suggesting a mechanism for estradiol in transcriptional regulation of DDAH2. In HUVECs, estradiol increased DDAH2 protein expression, decreased ADMA concentrations, and increased NO production (233); these effects could be blocked by ER antagonists (233, 234). Although not yet known, this regulation of DDAH2 by estradiol and ER may play an important role in cancers driven by excessive ER signaling, such as those of the breast.

## Impact of DDAH Expression on Tumor Angiogenesis and Vasculogenic Mimicry

A key aspect of cancer progression involves tumor angiogenesis. In addition to providing blood flow and nutrients to the tumor to support growth, angiogenesis is also implicated in tumor invasion and metastasis as the vasculature provides the tumor with access to distant organs. This is of particular concern when vasculogenic mimicry (VM), the process in which vascular-like structures are generated by cancer cells, is present. These vascular-like structures are not only able to fuse to the conventional vascular network (16), but they can remodel the vasculature such that it becomes “leaky” (235). Several studies including our own have demonstrated the functional role that increased DDAH expression has on both tumor angiogenesis and VM.

To the best of our knowledge, the only study to assess the role of DDAH2 on tumor angiogenesis was undertaken in lung adenocarcinoma. In surgically resected specimens, high expression of DDAH2 in stroma of invasive lung adenocarcinoma correlated with stronger eNOS expression in the vascular endothelium of the malignant tissue (166). *In vitro* assessment of recombinant DDAH2 expression in HUVECs demonstrated a significant increase in cell proliferation and capillary-like tube formation, in a model of angiogenesis (166). Whilst together these findings may be indicative of a model whereby DDAH2 promotes tumor angiogenesis, a more definitive assessment of the role of DDAH2 *in vivo* is clearly required.

To study the effect of DDAH1 on tumor growth and vascular development, Kostourou and colleagues generated a rat C6 glioma cell line over-expressing the rat DDAH1 isoform (236–239). The increased DDAH1 expression resulted in increased NO synthesis, as indicated by increased cGMP production, combined with increased expression and secretion of VEGF. Whilst no change in cell proliferation was observed in *in vitro* assays, DDAH1 overexpressing cells grew approximately two-fold faster than wildtype cells following subcutaneous injection into the flanks of nude mice (236). The use of non-invasive magnetic resonance imaging (MRI) for the assessment of blood vessel development *in vivo* demonstrated significantly increased vascularity in these tumors; this was further supported by increased tumor perfusion as assessed by Hoescht 33342 staining of functionally perfused vessels. It is thus plausible that the increased growth of DDAH1 overexpressing tumors is a direct result of increased blood vessel development. Further analysis of the tumor angiogenesis identified no difference between vascular maturation, vascular function and microvessel size between wildtype and DDAH1 overexpressing cells, suggesting a role for DDAH1 in the initial stages of vasculogenesis (237). Collectively, these studies were the first to demonstrate the importance of DDAH1 in regulation of tumor vessel development and clearly demonstrated that DDAH1 expression leads to more hypoxic tumors, higher blood volume, better tumor perfusion, and increased number of functional vessels (236–238).

It has been further demonstrated that xenografts derived from cells over-expressing an active site DDAH1 mutant (incapable of metabolizing ADMA) display an intermediate phenotype between tumors overexpressing wildtype DDAH1 and control tumors in terms of growth rate, endothelial content (vessel area), and hypoxia (239). However, VEGF production by inactive DDAH1-expressing cells is not significantly altered compared to wildtype cells (239). Thus, it appears that whilst DDAH1 metabolic activity is essential for the change in VEGF production (236, 239), cell growth and tumor vascularity are not entirely dependent upon ADMA metabolism and VEGF production. One hypothesis put forth by Boult et al. is that metabolically inactive DDAH may still be able to bind and hold ADMA, thus sequestering it away from NOS and relieving NOS inhibition (239). Further support for this hypothesis is provided by an elegant study in which DDAH1 was overexpressed under control of a pTet-Off regulatable element in rat C6 glioma cells deficient in NO production. Xenografts derived from cells with DDAH1 overexpression, lacking the ability to produce NO, were not significantly different in terms of size, vessel density, vessel function, or vessel maturation when compared to cells absent for DDAH1 expression and NO function (240). Together these studies suggest that, at least in C6 gliomas, the effect of DDAH1 on tumor growth and angiogenesis is purely NO-dependent.

In prostate cancer cell lines, exogenous expression of human DDAH1 increases cell proliferation, migration and invasion, and induces expression of multiple NO-regulated genes such as VEGF, HIF-1α, and iNOS. In alignment with the studies in rat C6 glioma cells, inhibition of NOS by L-NAME or 1400 W is sufficient to reverse the induction of all three pro-angiogenic genes. Furthermore, overexpression of an active site mutant human DDAH1 does not significantly alter cell behavior or VEGF expression, providing additional evidence that hydrolytic activity of DDAH1 is required for mediation of prostate cancer growth. Similarly, *in vivo* assessment of mouse xenografts has demonstrated significantly increased tumor size, invasion into muscular regions, mitotic figures, necrosis, pro-angiogenic factor expression, and tumor microvessel number in wildtype DDAH1-overexpressing tumors compared to mutated DDAH1-overexpressing and control tumors (54).

In our own studies assessing VM in triple negative breast cancer cell lines, specific knockdown of endogenous DDAH1 significantly attenuated cell migration, but not proliferation. Formation of vessel-like networks in an *in vitro* assay of VM, and VEGF expression, were also significantly reduced (187). Interestingly, expression of a miR-193b mimic, a direct negative regulator of DDAH1, completely abolished vascular channel formation (187); this is perhaps suggestive of miR-193b regulating a network of genes involved in VM. In contrast, exogenous expression of DDAH1 in a DDAH1-null breast cancer cell line was not sufficient to induce VM (187), indicating that DDAH1 is required but not sufficient for VM in breast cancer. The extent to which DDAH1 can modulate breast cancer VM via ADMA-dependent or -independent processes is yet to be established.

### Pharmacological Inhibition of DDAH1 Activity in Cancer

There are currently no synthetic compounds which act as specific DDAH1 or DDAH2 activators, nor are there any selective DDAH2 inhibitors. With the exception of a few compounds specifically targeting bacterial DDAH (241–243), all other synthetic DDAH inhibitors have been synthesized to selectively target DDAH1. Despite enhanced DDAH2 expression being linked to a handful of cancers such as lung (166) and prostate (184), the lack of a robust and reproducible *in vitro* DDAH2 activity assay represents a significant limitation for the development and pharmacokinetic characterization of DDAH2 activity modulators. As a consequence, studies investigating the effects of DDAH pharmacological inhibition focus solely on DDAH1. Over the last two decades various different classes of DDAH1 inhibitors have been synthesized; these exhibit different structures, features and mechanisms of action, and have been previously extensively reviewed (244). Whilst some of these molecules have structural similarity with the DDAH substrates (methylated arginines) (183, 245–249), others bear a very different chemical structure (56, 250–252). A comprehensive discussion on all DDAH inhibitors synthesized to date and their impact on endothelial cells falls outside the scope of this review, however, here we summarize a small body of evidence that identifies the therapeutic potential for pharmacological inhibition of DDAH1 in cancer.

The first study to show some potential for DDAH1 inhibition by a small molecule in cancer was published by (183). The research group demonstrated that DDAH1 is overexpressed in melanoma cell lines compared to normal human epidermal melanocytes and that cellular inhibition of DDAH1 by *N*^5^-(1-imino-2-chloroethyl)-L-ornithine (Cl-NIO) resulted in reduced nitric oxide production in the A375 melanoma cell line. The reduction in NO synthesis was measured by quantifying 3-nitrotyrosine levels and total nitrate and nitrite in the cell culture supernatant and it was independent of changes in DDAH1 or iNOS expression (183). Unfortunately, this study did not assess the effects of DDAH1 inhibition by Cl-NIO on specific tumor parameters, such as tumor cell viability and proliferation *in vitro* and/or *in vivo* growth of xenograft tumors derived from A375 cells, or assess the impact on angiogenesis.

More recently, the potential therapeutic benefit of inhibiting DDAH1 was demonstrated for breast cancer (55). DDAH1 activity was inhibited in triple negative breast cancer cell lines by the potent DDAH1 inhibitors, arginine analogs ZST316 and ZST152 (244, 249), as identified by increased intracellular ADMA concentrations and decreased intracellular L-citrulline concentrations (55). In an *in vitro* Matrigel tube formation model of VM, both ZST316 and ZST152 significantly inhibited the number of vessel-like networks formed at concentrations above 1 μM (55). Importantly, the endogenous NOS inhibitor L-NMMA, which is widely used as a tool to decrease NO availability, also significantly reduced tube formation in these assays. By contrast, no inhibition was observed when cells were treated with SDMA, which is neither a substrate for DDAH1 nor an inhibitor of NOS. Cell viability and proliferation were not affected by doses of up to 100 μM of ZST316 or ZST152, however, a decrease in cell migratory potential was observed, which may be in part responsible for the reduced tube formation in the model of VM (55). Although, these results are somewhat preliminary and need further confirmation with *in vivo* studies, they suggest a promising role for DDAH1 inhibition as a novel treatment strategy in triple negative breast cancer.

The most recent and comprehensive study which describes a role for DDAH1 pharmacological inhibition in cancer demonstrates the ability of the compound 3-amino-6-tert-butyl-N-(1,3-thiazol-2-yl)-4-(trifluoromethyl)thieno[2,3-*b*]pyridine-2-carboxamide (DD1E5) to irreversibly inhibit DDAH1 activity in prostate cancer cells (56). Treatment with DD1E5 inhibited proliferation, migration and invasion of prostate cancer cell lines LNCaP and PC3, but was also able to attenuate proliferation of cells stably overexpressing DDAH1; this was accompanied by decreased DDAH1 enzymatic activity, increased ADMA concentration and decreased NO synthesis. Additionally, modulation of the angiogenic pathway was observed in prostate cancer cells following treatment with DD1E5: the pro-angiogenic factors VEGF, iNOS, c-Myc, and HIF-1α were all downregulated, indicating that DDAH1 inhibition attenuates the angiogenic potential of DDAH1+ cells (56). The release of pro-angiogenic signals bFGF and IL8 was also decreased following DDAH1 inhibition, and this translated into a decrease in endothelial cell tube formation when cells were cultured in conditioned media from the treated prostate cancer cells. Most importantly, *in vivo* analysis demonstrated that DD1E5 inhibited the growth of xenograft tumors derived from DDAH1 overexpressing PC3 cells, reduced the expression of VEGF, NOS, and HIF-1α in xenograft tumors, and resulted in poorer vascularization as assessed by micro vessel density (56).

## Conclusion

The DDAH enzymes are responsible for the metabolism of the endogenous NOS inhibitors, the asymmetrically methylated arginines ADMA and L-NMMA, and are thus critical factors in both maintaining and modulating precise NO production. In endothelial cells, the significance of the DDAH/ADMA/NO axis is well-documented: NO has a regulatory role which is required for endothelial cell activation, proliferation and migration, and which overall is necessary for effective angiogenesis. Studies have consistently demonstrated that dysregulation of this pathway and NO synthesis, as a consequence of DDAH modulation, results in impaired angiogenesis (142, 149, 164, 253).

The role of NO has been extensively studied in cancer, particularly tumor angiogenesis, yet the literature is not always entirely clear. It appears that NO can have both oncogenic and protective roles depending on cancer type, location and stage, as well as local NO concentration and exposure duration. Nonetheless, excessive NO production has been associated with poor prognosis, increased vasculature and increased invasiveness of multiple cancers such as breast (102, 104, 105), prostate (107), and colorectal (110, 111). Until recently, the majority of studies which have assessed the impact of altered NO production in cancer have focused solely on the role (both expression and regulation) of the three NOS enzymes. In contrast, limited studies have addressed the potential impact of DDAH expression and function in the oncology setting. As discussed here, DDAH expression (particularly DDAH1) is significantly altered in a number of different cancers. In the majority of these, DDAH expression is increased and is associated with increased NO concentrations, increased VEGF expression and increased cell aggressiveness. Furthermore, *in vivo* studies using DDAH1 overexpression models have demonstrated increased tumor growth and corresponding increased tumor vasculature. Whilst one of the roles of DDAH1 in tumor vessel development is likely facilitation of endothelial cell migration and invasion, as supported by DDAH1 overexpression conditioned media studies (236), initial reports in breast cancer cell lines suggest that DDAH1 is also a modulator of VM. Whether the function of DDAH1 in VM is entirely ADMA/NO-dependent remains to be determined. In contrast to DDAH1, the importance of DDAH2 in ADMA metabolism and thus tumor angiogenesis is still unclear. Collectively, these studies begin to further elucidate the complex tumor-promoting pathways in multiple cancers.

Importantly, the upregulation of DDAH1 expression and consequent increased enzymatic activity may suggest a novel role for DDAH1 in tumor progression, providing novel diagnostic, and therapeutic opportunities for DDAH1 as a possible molecular drug target. Intriguingly, DDAH1 autoantibodies have been detected in sera of prostate cancer patients and proposed as a new marker for a novel prostate cancer and benign hyperplasia diagnostic, improving on the traditional prostate specific antigen (PSA) test which often yields false-positive results (254). The exact mechanism responsible for production of DDAH autoantibody markers is unknown but may relate to changes in DDAH1 expression levels. A handful of studies have assessed the impact of DDAH1 inhibition by small molecules in cancer with promising results for inhibition of tumor growth, vasculature density, and VM. Taken together, they demonstrate that pharmacological inhibition of DDAH1 represents a novel, alternative strategy for the treatment of cancers associated with elevated DDAH1 expression and activity. Studies on breast cancer, prostate cancer, glioma, and melanoma have identified that these cancers typically express high levels of DDAH1 and are dependent on DDAH/ADMA/NO signaling for cell survival, proliferation, migration, and/or angiogenesis; these cancers would be prime candidates for treatment by DDAH1 inhibition. It is currently unknown as to whether DDAH1 inhibitors act exclusively by blocking enzymatic activity or whether they may modulate alternative functions of DDAH1 (e.g., potential protein-protein interactions).

Although studies are limited, the data to date suggests a basis for the development of DDAH1 inhibitors to be used as combined anti-angiogenic and anti-VM agents in cancer. It is important to continue to unravel the mechanisms of DDAH1-mediated tumor angiogenesis and VM, and to further explore the potential of selectively inhibiting DDAH1 activity across different tumor types and stages. Pending the results of animal studies, the use of DDAH1 inhibitors, alone or in combination with traditional anti-angiogenic therapies such as anti-VEGF drugs, might represent a novel strategy to suppress both angiogenesis and VM, key factors in early cancer development and dissemination. Based on the current evidence, which highlights the lack of a clear direct cytotoxic effect of small molecule DDAH1 inhibitors, it would appear that the best therapeutic window is within the early stages of cancer development, typically driven by neovascularization, in order to timely prevent dissemination and metastasis. This, however, does not exclude their potential use in the later stages of the disease, particularly if combined with other pharmacological strategies. Given the importance of DDAH1 in maintaining homeostasis of the cardiovascular system, particularly in attenuating cardiovascular disease and heart failure, potential negative impacts of inhibition of DDAH1 must be considered. As such, further studies are required to determine whether DDAH1 inhibitors can be safely administered systemically or whether approaches for a targeted, local, delivery are preferable.

## Author Contributions

J-AH and ST wrote the first draft of the manuscript and prepared figures. EG, NJ, RR, and AM contributed to manuscript revision, read, and approved the final submitted version.

### Conflict of Interest

The authors declare that the research was conducted in the absence of any commercial or financial relationships that could be construed as a potential conflict of interest.
